# Post-traumatic growth and religious coping in Muslims exposed to the March 15 terror attacks in New Zealand: A cross-sectional study

**DOI:** 10.1177/00048674251374461

**Published:** 2025-09-16

**Authors:** Ben Beaglehole, Joseph M Boden, Richard Porter, Ruqayya Sulaiman-Hill, Fareeha Ali, Shaystah Dean, Zeenah Adam, Sandila Tanveer, Philip J Schluter, Caroline Bell

**Affiliations:** 1 Department of Psychological Medicine, University of Otago, Christchurch, New Zealand; 2 Private Practice, New Zealand; 3 School of Health Sciences, Faculty of Health, University of Canterbury, Christchurch, New Zealand; 4 General Practice Clinical Unit, Faculty of Medicine, The University of Queensland, Brisbane, QLD, Australia

**Keywords:** Post-traumatic growth, religious coping, March 15 shootings, trauma

## Abstract

**Objective:**

On March 15, 2019, a white supremacist attacked two mosques in Christchurch, New Zealand causing 51 deaths. The March 15 study was initiated to evaluate the mental health and wellbeing of the affected Muslim community. Given participants shared faith and reported growth through adversity, an evaluation of post-traumatic growth and religious coping was included.

**Methods:**

The March 15 study assessed sociodemographic and clinical factors 11–32 months after the shootings. The Post-Traumatic Growth Inventory and the Religious Coping Scale were administered. Descriptive statistics were used to report the presence of moderate-high post-traumatic growth. Bivariable analyses between Post-Traumatic Growth Inventory and potential predictor variables were undertaken. Measures that were significantly (*p* < 0.05) or marginally (*p* < 0.10) associated with post-traumatic growth were included in a multivariable regression model.

**Results:**

Data for 187 participants were analysed. Fifty-eight percent of the participants met criteria for moderate-to-high post-traumatic growth. Being present at a mosque during the shootings (*p* = 0.011) and use of a life coach after the shootings (*p* = 0.02) were positively associated with post-traumatic growth. Family tensions and holding a university degree were negatively associated with post-traumatic growth (*p* = 0.035, 0.011). When the Religious Coping Scale was included in the multivariable model, it explained a large proportion of the variance associated with post-traumatic growth (*p* < 0.001).

**Conclusion:**

The participants experienced relatively high levels of post-traumatic growth. The dominance of religious coping in the multivariable model suggests that the Post-Traumatic Growth Inventory and Religious Coping Scale assessed similar qualities for Muslims impacted by the March 15 shootings. Qualitative research is required to broaden understanding of the underpinnings to post-traumatic growth.

## Introduction

On March 15, 2019, a white supremacist attacked two mosques during Friday prayers in Christchurch, New Zealand, resulting in 51 deaths and numerous injuries (Sulaiman-Hill et al., 2021). Following the terrorist attack, there were widespread concerns about the physical and mental health of those affected. In order to better understand these concerns, the March 15 study was conceived (Sulaiman-Hill et al., 2021). This is a mixed-methods study incorporating culturally acceptable self-report measures and clinician-administered diagnostic interviews. The study is intended to be longitudinal although only the first-phase of data collection has occurred, 11–32 months after the shootings.

Initial findings from the study reported that 61% of the study population experienced at least one mental disorder following the attacks including anxiety, depression, and post-traumatic stress disorder (Bell et al., 2024). Eighty percent of the affected community had been exposed to major traumatic events prior to the shootings, including the Canterbury earthquake sequence (Beaglehole et al., 2019), and many faced a range of stressors subsequent to the shootings (Sulaiman-Hill et al., 2024). Despite adverse consequences of the shootings, religious coping and post-traumatic growth (PTG) were highlighted following the attacks (Sulaiman-Hill et al., 2024). In addition, the qualitative arm of the study reported that one of the central themes of the post-shootings experience was gaining meaning and growth, with Islamic faith being central to this position (Dean et al., 2024).

PTG refers to the positive psychological changes that may occur when individuals face highly challenging life circumstances (Linley and Joseph, 2004). An assessment of PTG is important to achieve a holistic understanding of the impacts of trauma. The Post-Traumatic Growth Inventory (PTGI) is the most widely used tool to measure PTG (Tedeschi and Calhoun, 1996). The PTGI is designed to measure changes over five domains of life (‘relating to others’; ‘new possibilities’; ‘personal strength’; ‘spiritual change’; and ‘appreciation of life’), following the experience of highly stressful life events. The PTG literature includes reporting on the internal and external factors associated with PTG for individuals or groups exposed to adversity, including exposure to terrorist attacks (Fausor et al., 2022; Ikizer and Ozel, 2021). Findings include the presence of positive associations between PTG and cumulative trauma after the attack (Fausor et al., 2022) and between resilience and PTG (Ikizer and Ozel, 2021).

Fayaz (2023) evaluated the role of religion in PTG after armed conflict. They identified key themes including a positive association between PTG and religiosity, and religious coping as a source of PTG. Religious coping is the use of religion to manage stress. Much of the religious coping literature evaluates Christian populations living in Western countries so the 24-item Muslim Religious Coping scale (MRC) was developed to quantify religious coping for Muslim participants living in the context of Islamophobia in New Zealand (prior to the March 15 shootings) (Adam and Ward, 2016). The MRC evaluates subdomains of cognitive, behavioural, and social religious coping.

### Study aim

We aim to highlight PTG and religious coping as part of the trauma-response in those exposed to terrorist attacks and allow comparisons to be made with similar populations. We therefore report PTGI scores and factors associated with PTG for Muslims exposed to the March 15 shootings. We also report findings from an adapted MRC to consider the relationship between PTG and religious coping.

## Methods

### The March 15th study

The March 15th Study protocol is registered with the Australian New Zealand Clinical Trials Registry (ACTRN12620000909921). The Strengthening the Reporting of Observational Studies in Epidemiology (STROBE) guidance for observational cross-sectional studies checklist was used to guide reporting (see Supplementary Material for details) (Von Elm et al., 2014).

### Ethics

The March 15 Study was approved by the New Zealand Health and Disability Ethics Committee (HDEC Reference 19/NTA/147). Conduct of the study complied with ethical standards for human experimentation established by the Helsinki Declaration of 1975, as revised in 2008, and was performed in accordance with the ethical standards from HDEC. All participants provided written informed consent. Participants were free to withdraw at any time without penalty.

### Design

The study employed a mixed-methods design including a clinician-administered clinical interview and diagnostic assessment, self-report measures, and a qualitative substudy (Dean et al., 2024; Sulaiman-Hill et al., 2021). As no official list was available of those present in the mosques at the time of the attacks, recruitment relied on community engagement and promotion of the project. Of the 201 individuals who contacted the study team, 12 were ineligible or failed to complete the assessments, leaving a study population *n* = 189 participants.

Interviews of the 189 participants took place between February 2020 and December 2021 (11–32 months after the attacks). Initial inclusion criteria were being an adult Muslim living in the Christchurch region from one of the following groups: survivor present at or near either mosque during the attacks, close relative of one of the 51 people who died or close relative of someone from the survivor group. Following feedback from the community, the eligibility criteria were expanded to include all adult members of the Christchurch Muslim community present in the city when the attacks occurred and at the time of interview. This community totals approximately 4000 Muslims including many children. Exclusion criteria included living outside Christchurch at the time of recruitment and aged under 18 years.

### Measures

#### Post-Traumatic Growth Inventory

The PTGI is the primary outcome and was used to assess PTG (Tedeschi and Calhoun, 1996). This is a 21-item scale that evaluates five domains (relating to others, new possibilities, personal strength, spiritual change and appreciation of life) following a stressful life-event. Participants respond to each item by rating change scores from 0 (‘no change’) to 5 (‘I experienced this change to a very great degree as a result of my crisis’) allowing a maximum PTG score of 105. The PTGI has good internal consistency (α = 0.90) (Tedeschi and Calhoun, 1996).

#### Religious Coping Scale

The MRC (Adam and Ward, 2016) was modified for use in the March 15 study after consultation with the scale developer. An open-ended question specific to the March 15 experience was added (Are there other ways that you have used your faith to help you cope since March 15?), question 14 was changed to be anchored to the time period following March 15, and the scale was shortened to reduce participant fatigue. The revised MRC is titled the Religious Coping Scale (RCS). The RCS is a 14-item scale that measures religious coping for Muslims across cognitive, behavioural and social domains. The cognitive religious coping domain measures the use of reasoning to make sense of divine motives in traumatic events; for example, seeking patience because Allah is with those who are patient. Behavioural religious coping measures religious behaviours such as making dua (supplications) and reading the Qur’an. Social religious coping measures activities such as attending events in the Muslim community or looking to the community for love and concern. Each item is scored on a five-point Likert-type scale with greater numbers recording the extent to which participants engaged in a particular coping domain. In the original MRC, the scale demonstrated strong internal reliability for the overall measure of Muslim Religious Coping (α = 0.94), as well as for each MRC subscale (Cognitive α = 0.92; Behavioural α = 0.92; Social α = 0.85) (Adam and Ward, 2016). Cronbach’s alpha for the RCS = 0.93.

#### Exposure

The extent of exposure to the mosque attacks was assessed by asking participants to respond to as many items as were relevant, including being a member of the wider Muslim community living in Christchurch at the time of the attacks, being present at one of the mosques during the attacks, being a family member of someone present during the attacks, being bereaved by the attacks, and being a family member of someone injured during the attacks. Participants could be recorded in multiple exposure categories (Bell et al., 2024).

#### Covariate factors

Potential covariate factors considered in the analysis included sociodemographic variables such as age, gender, ethnicity, English language proficiency, level of education, and prior trauma exposure that were assessed by self-report. Measures of whether participants were experiencing any of a range of potential stressors such as financial concerns, housing problems, immigration stress, employment issues, family tensions, and concern about family were chosen on the basis of the literature on exposure to traumatic stress. Study members were also asked about their participation in a range of health promoting activities and utilisation of health and social services, that were specifically provided for families and survivors of the shootings by local and central government. The health promoting activities included attendance at Muslim social events, Islamic scholar meetings, Life coach services, Māori community events and parent workshops. Social services included Government disability and financial support, Purapura Whetu (a Māori support service), Victim support services, the Police family liaison service, Secondary care physical and mental health services, and Immigration services.

The presence of anxiety disorder, major depressive disorder, or post-traumatic stress disorder (PTSD) prior to the attacks, following the attacks, and at the time of interview was assessed using the semi-structured interview, Mini-International-Neuropsychiatric-Interview (MINI) (Sheehan et al., 1998). Psychological distress was measured by the Kessler-10 (K-10) (Kessler et al., 2003). Post-traumatic stress disorder was also assessed using the PTSD checklist (PCL-5) (Weathers et al., 2018). Full details of potential covariates are in the original protocol paper and initial outcome paper (Sulaiman-Hill et al., 2021, 2024).

## Statistical analysis

Descriptive statistics were used to report total and subscale PTGI and total scores. The prevalence of moderate-to-high PTG (using the definition provided by Wu et al. of scoring ⩾ 63 on the PTGI (Wu et al., 2019)) was calculated to enable comparisons with other study populations. For the RCS, the mean total score is reported as well as the mean score for items within the subdomains as the subdomains have differing numbers of items. There were only two participants with missing data and these were deleted list-wise from the analysis.

In order to examine the predictors of PTGI, in the first step of the analyses, Pearson product moment correlations (for continuous measures) and Spearman correlations (for categorical or dichotomous measures) were used to determine the set of independent variables that were most strongly associated with the PTGI measure. The independent variables were post-March 15 mental health disorders; individual factors; membership in one or more exposure groups (level of exposure to the attack); reports of family stressors; post-March 15th activities; and services accessed post March 15th. Those measures that were statistically significantly (*p* < 0.05) associated with PTG, or marginally (*p* < 0.10) associated with PTG, were retained for multivariable analyses.

In the next step of the analyses, we fitted a multivariable linear regression model (Model 1) with the continuous PTGI score as the dependent variable, and the independent variables identified in the previous step (using Pearson/Spearman correlations). Variables were entered into the regression equation using a forward and backward stepwise analysis package (Stata ‘stepwise’) in Stata 18.5 (StataCorp, College Station, TX), using robust standard errors to reduce the influence of outliers. In ‘stepwise’, variables were retained only if their adjusted p-value did not exceed 0.05. The variables ‘age’, ‘gender’ and ‘previous trauma exposure’ were included in final modelling irrespective of p-value, to ensure that the model was adjusted for gender, age and previous trauma exposure.

In the final step of the analyses, we extended the model predicting PTGI to include the measure of the RCS as an independent variable (Model 2). This variable had been omitted previously due to a very strong correlation with PTGI (0.48). Models were checked for heteroscedasticity, multicollinearity, and the assumption of a normal distribution of the dependent variable.

Finally, as a sensitivity analysis, the regression models described above were modified, so that the outcome measure was a dichotomous variable corresponding to whether the person scored ⩾63 on the PTGI total scale score, and logistic regression analyses were undertaken using the same set of predictors. The results of this analysis closely matched those reported below.

## Results

There are 189 participants in the March 15 study of which 187 completed the PTGI and are included in this analysis. The mean PTGI for these participants was 65.4 (SD = 23.0). The mean subscale scores were: ‘Relating to others’ 21.2 (SD = 8.1), ‘New possibilities’ 13.7 (SD = 6.3), ‘Personal strength’ 13.3 (SD = 5.3), ‘Spiritual change’ 6.9 (SD = 3.0), and ‘Appreciation of life’ 10.5 (SD = 3.7). Fifty-eight percent of the participants (*n* = 109) scored ⩾63 on the PTGI indicating moderate-to-high PTG for this group.

The mean overall score of the RCS was 49.8 (SD = 13.9) and the mean subscale item scores for cognitive religious coping, behavioural religious coping, and social religious coping subscales were 4.07 (SD = 1.2), 3.78 (SD = 1.3), and 2.63 (SD = 1.1), respectively.

### Bivariable analysis

Table 1 shows the mean PTGI scores and standard deviations for group membership status (yes/no). These associations were tested for significance using the chi-square test of independence. Only the PTGI scores and measures with statistically significant (*p* < 0.05) or marginally significant (*p* < 0.10) associations are reported in the table.

**Table 1.** Post-traumatic growth scores by group membership^a^.

	Yes			No			p^a^
	N	Mean	(SD)	N	Mean	(SD)	
**Individual factors**							
“Very good” spoken English proficiency	86	63.64	(25.12)	101	67.27	(20.80)	0.020
University degree holder	108	61.88	(23.40)	79	70.68	(21.31)	0.010
**Exposure group**							
At mosque	41	72.78	(22.35)	146	63.58	(22.72)	0.023
Family present, not injured	36	73.28	(24.55)	151	63.77	(22.18)	0.025
Family present, injured	66	70.29	(21.47)	121	63.04	(23.34)	0.038
**Reports of Family Stressors**							
Family tensions	53	59.98	(21.92)	133	67.77	(23.05)	0.036
Immigration issues	29	61.00	(14.11)	157	66.39	(24.17)	0.060
**Activities post-March 15** ^**th**^							
Māori community events attendance	33	73.15	(22.64)	154	63.98	(22.70)	0.036
Muslim social event attendance	138	67.30	(22.16)	49	60.80	(24.47)	0.087
Islamic scholar attendance	89	69.08	(21.38)	98	62.44	(23.86)	0.047
Life coach	24	75.42	(17.24)	163	64.15	(23.31)	0.024
Parent workshop	27	73.11	(21.45)	160	64.33	(22.95)	0.065
**Services post-March 15** ^**th**^							
Government disability and financial support	92	69.65	(21.23)	95	61.67	(23.87)	0.017
Purapura Whetu (Māori support services)	20	75.35	(23.32)	167	64.43	(22.63)	0.044
Victim Support services	85	68.95	(22.14)	102	62.80	(23.25)	0.067
Police family liaison service	66	71.42	(19.58)	121	62.42	(24.00)	0.010
Secondary care physical health services	29	75.14	(20.48)	158	63.85	(22.94)	0.014
Secondary care mental health services	20	74.65	(24.87)	167	64.52	(22.49)	0.061
Immigration services	54	70.52	(21.30)	133	63.60	(23.30)	0.061

a *P* value derived from chi-square test of independence.

Those participants who self-reported their spoken English as ‘very good’, and who held a university degree or equivalent, had significantly lower PTGI scores than participants who did not report these (*p* = 0.02, 0.01, respectively).

Significantly higher PTGI scores were present for the following exposure groups: present at the mosques (*p* = 0.023), family member present (but not injured) at the mosques (*p* = 0.025), and family member present at the mosques and injured (*p* = 0.038).

Significantly lower PTGI scores were present if family tensions were present after the March 15 shootings (*p* = 0.036).

Undertaking the following activities following the shootings were associated with significantly higher PTGI scores: Māori community events attendance (*p* = 0.036), Islamic scholar attendance (*p* = 0.047), and utilising a life coach (*p* = 0.024).

Utilising the following services were also associated with significantly higher PTGI scores: Government disability and financial support (*p* = 0.017), Purapura Whetu (Māori support services) (*p* = 0.044), Police family liaison support (*p* = 0.010), and secondary care physical health services (*p* = 0.014).

Correlations between PTGI total score and independent variables are presented in Supplemental Table S1. In addition, the correlations between PTGI and three independent variables were as follows: gender; *r* = 0.056 (*p* = 0.49); age at the time of assessment; *r* = 0.008 (*p* = 0.91); and previous traumatic experiences score; *r* = –0.044 (*p* = 0.55). Although none of these variables were significantly associated with PTG, they were carried forward in the multivariable analysis reported below to account for potential effects of gender, age and past trauma on the multivariable models. The Pearson correlation between PTGI and RCS was 0.48 (*p* < 0.001).

### Multivariable analyses

The tests of model assumptions revealed no evidence of heteroscedasticity or multicollinearity among the independent variables. For multivariable Model 2, there was significant (*p* < 0.05) kurtosis in the distribution of PTG.

Tables 2 and 3 show the parameter estimates, standard errors and p-values for the multivariable models predicting PTG among the March 15th cohort. P-values were derived from t-tests in ordinary least squares regression. Table 2 (Model 1) shows that being present at a mosque during the shootings (*p* = 0.015) and use of a life coach after the shootings (*p* = 0.017) remained positively associated with PTG in the multivariate model. University degree holder and family tensions were negatively associated with PTG (*p* = 0.011, 0.039, respectively). No other independent variables were found to be significantly associated with PTG in Model 1. Beta-scores ranged from -0.18 (University degree holder) to 0.20 (at Mosque during attack). The *R*^2^ value for Model 1 was 0.12.

**Table 2.** Fitted regression model of PTGI scores and independent variables (Model 1).

	B	SE	95% CI	P^a^	β
Gender (female)	5.52	3.88	–2.14 – 13.19	0.157	0.12
Age	–0.06	0.13	–0.32 – 0.20	0.671	–0.03
Previous trauma score	–0.69	1.62	–4.59 – 3.20	0.726	–0.03
University degree holder	–8.32	3.25	–14.73 – –0.37	0.011	–0.18
At mosque during attack	10.72	4.34	2.60 – 20.01	0.015	0.20
Life coach attendance	10.06	4.17	1.82 – 18.30	0.017	0.15
Family tensions present	–7.60	3.66	–14.84 – –0.37	0.039	.15

a *P* value derived from *t* test from ordinary least squares regression model.

**Table 3.** Fitted regression model of PTGI scores and predictors after adding RCS (Model 2).

	B	SE	95% CI	p^a^	β
Gender (female)	0.57	3.0	–5.36 – 6.50	0.850	0.02
Age	–0.09	0.14	–0.73 – 0.19	0.518	–0.04
Previous trauma score	0.22	1.62	–2.98 – 3.41	0.893	.01
RCS total score	0.73	0.13	0.47 – 0.99	< 0.001	0.45
University degree holder	–6.99	3.02	–12.95 – –1.03	0.022	–0.15
Involved with police-family liaison	10.37	3.48	3.43 – 17.18	0.022	0.21
Involved with victim support services	–7.21	3.57	–14.27 – –0.15	0.045	–0.15

a *P* value derived from *t* test from ordinary least squares regression model.

When the RCS was included in the multivariate model (Table 3, Model 2), RCS score was positively associated with PTG (*p* < 0.001), as well as accessing the Police Family liaison service (*p* = 0.004). Both University degree holder (*p* = 0.022) and accessing victim support services (*p* = 0.045) were associated with lower PTG scores. No other independent variables were found to be associated with PTG in multivariable Model 2. Beta-scores ranged from -0.15 (University degree holder and involved with victim support services) to 0.45 (RCS total score). The *R*^2^ value for Model 2 was 0.27.

## Discussion

The Muslim community affected by the March 15 shootings are ethnically and demographically diverse but united by exposure to terrorist shootings and shared Muslim faith while living in a Western country. Despite their trauma, we expected to observe PTG following the shootings given literature reporting PTG in diverse settings. We hypothesised that religious coping would be a key feature. We therefore completed this study to quantify personal growth after the shootings and highlight positive consequences of trauma. We aimed to better understand how PTG arose and its interplay with religious coping.

The mean PTG score in our study population was 65.4 (SD = 23.0) and 109 (58.2%) participants were classified in the moderate-to-high PTG category. Other studies evaluating PTG following terrorist attacks are rare but the mean PTGI score 30 years following terrorist attacks in Spain was 41.78 (SD = 27.32) (Fausor et al., 2022) and the prevalence of moderate-to-high PTG ranged from 10% to 77.3% in a systematic review of populations exposed to a range of traumas (Wu et al., 2019). Our findings therefore suggest the March 15 survivors experienced relatively high levels of PTG compared with other populations exposed to trauma.

In the review by Wu et al. (2019), moderate-to-high PTG was more likely in younger populations (aged <60 years), in specific professions (such as firefighters or veterans), when PTG was evaluated within 6 months of the trauma, and following direct exposure to the trauma. In our analysis, prior to including the RCS in the multivariate analysis, using a life coach after the shootings and being present at one of the two mosques during the shootings were positively associated with PTG. The presence of family tensions after the shootings and being a university degree holder were negatively associated with PTG. Regression coefficients ranged from –0.15 to 0.20 suggesting that the independent variables that significantly predicted PTG in the model had similar effect sizes and were relatively small in magnitude.

When the RCS was included in the multivariate analysis, religious coping was the largest factor associated with PTG. One possibility for the dominance of religious coping in the explanatory model is that the PTGI and RCS are assessing similar qualities for Muslims impacted by the March 15 shootings. A previous study evaluated positive and negative religious coping and PTG among Muslims who lost their children due to traffic accidents (Abu-Raiya and Sulleiman, 2021). This study used the 14-item Brief RCOPE to measure religious coping; the Brief RCOPE includes positive items (e.g. Sought God’s love and care) and negative items (e.g. Wondered whether God had abandoned me) (Pargament et al., 1998). Abu-Raiya and Sulleiman (2021) concluded that positive religious coping and PTG were likely to be inherently linked. We did not assess for negative religious coping but in Abu-Raiya and Sulleiman’s study, there was an indirect negative association between negative religious coping and lower PTG scores.

A contextual basis is needed to understand the positive consequences of trauma. Although the terrorist targeted a religious minority group, the shootings prompted an immediate widespread response of grief, love, and solidarity from Muslims and non-Muslims alike (Anwar and Sumpter, 2022). We hypothesise that a positive social milieu enables PTG even if the trauma is horrific. The affected Muslim community were united by their shared Islamic faith. It is possible that there are factors within the Islamic faith that promoted PTG despite the abhorrent nature of the terrorist acts faced by the Muslim community. The Shuhada (martyrs) killed by the terrorist are considered alive in an elevated position in heaven. This can provide comfort and solace to family members. In addition, there is the Islamic concept that individuals are only tested with what they can bear, as well as moral imperatives to assist others and make a positive contribution to the community. These factors could be influential in fostering resilience and promoting a sense of purpose for survivors.

The assessments of PTG and religious coping were completed 11–32 months after the shooting and the timing is likely to affect scores for these measures. Wu et al. (2019) reported that PTG was more likely when it was assessed within 6 months of the trauma. It is unclear if PTG and religious coping will fade or grow as the impacts of the March 15 shootings become more distant. Further assessments of the affected population are planned to ascertain the trajectory of PTG and religious coping over time.

### Strengths and limitations

A key strength of this study is that we considered positive as well as negative impacts of trauma by measuring PTG and religious coping. The comprehensive set of measures enabled a thorough assessment of the impacts of the March 15 shootings. These included a formal assessment of the community and government programmes provided to assist the March 15 recovery. The PTGI measures positive growth dimensionally. It does not provide standardised cut-points to designate PTG although we used thresholds designated by Wu et al. (2019) to report moderate-to-high PTG. We relied on the PTGI to quantify PTG. Other researchers have discussed the underlying nature of PTG, positing that PTG is utilised by individuals as a coping strategy to manage trauma. It may reflect positive affective, cognitive and behavioural change (Jayawickreme and Infurna, 2021) and encompass experiential spiritual engagement (Dean et al., 2024). This may capture what some Muslim conceptualise as a form of transcendence beyond the material world, whereby one’s spiritual heart (galb) is in harmony with the divine (Al-Ghazali and Winter, 2016). Understanding in more detail the nature of PTG is important. The cross-sectional nature of our current dataset means we cannot report whether PTG is stable over time or is associated with subsequent affective, cognitive or behavioural change. Further planned assessments may help understand the degree to which PTG in our sample was associated with improved mental, physical and functional outcomes longitudinally. In addition, we also note that the distribution of the PTG measure was significantly kurtotic, resulting in a distribution with very narrow ‘tailedness’. Attempts to rectify this via transformation were considered inappropriate as these transformed data tended to produce a bimodal distribution, and the analyses were undertaken with robust standard errors in an attempt to mitigate this problem. In addition, we recognise the quantitative nature of this study is not well-suited to explore complex personal experiences. We have planned complementary qualitative research to explore the in-depth experience of PTG and religious coping in this setting.

## Conclusion

We highlight the presence of PTG and religious coping following the March 15 shootings to ensure that both positive and negative consequences of trauma are acknowledged. The March 15 study population experienced relatively high rates of PTG compared with other trauma-exposed populations. Their shared Muslim faith was associated with religious coping and this was a dominant factor in the presence of PTG.

## Supplemental Material

sj-docx-1-anp-10.1177_00048674251374461 – Supplemental material for Post-traumatic growth and religious coping in Muslims exposed to the March 15 terror attacks in New Zealand: A cross-sectional study Supplemental material, sj-docx-1-anp-10.1177_00048674251374461 for Post-traumatic growth and religious coping in Muslims exposed to the March 15 terror attacks in New Zealand: A cross-sectional study by Ben Beaglehole, Joseph M Boden, Richard Porter, Ruqayya Sulaiman-Hill, Fareeha Ali, Shaystah Dean, Zeenah Adam, Sandila Tanveer, Philip J Schluter and Caroline Bell in Australian & New Zealand Journal of Psychiatry
