# Supplementary material for: Post-traumatic growth and religious coping in Muslims exposed to the March 15 terror attacks in New Zealand: A cross-sectional study
Source: Aust N Z J Psychiatry. 2025 Sep 16;59(11):1019–26. doi: 10.1177/00048674251374461 (PMC12569132; doi:10.1177/00048674251374461)
Supplement: sj-docx-1-anp-10.1177_00048674251374461 – Supplemental material for Post-traumatic growth and religious coping in Muslims exposed to the March 15 terror attacks in New Zealand: A cross-sectional study [file sj-docx-1-anp-10.1177_00048674251374461.docx]

Supplementary Table S1. Pearson/Spearman correlations between PTGI total score and independent variables

|  | Pearson/Spearman | |
| --- | --- | --- |
| Independent Variable | r | p |
| **Mental Health Disorders** |  |  |
| Post-Traumatic Stress Disorder post-March 15th | -0.023 | 0.75 |
| Major Depression Disorder post-March 15th | -0.100 | 0.18 |
| Anxiety Disorder post-March 15^th^ | 0.069 | 0.35 |
| **Individual Factors** |  |  |
| Time elapsed between March 15^th^ and participant interview | -0.087 | 0.24 |
| Participant gender | 0.056 | 0.49 |
| Participant age | 0.008 | 0.91 |
| Number of prior traumatic events reported | -0.044 | 0.55 |
| Marital status | 0.038 | 0.61 |
| Years living in New Zealand | -0.091 | 0.22 |
| Spoken English proficiency | -0.166 | 0.02 |
| Written English proficiency | -0.097 | 0.21 |
| Education level | -0.208 | 0.004 |
| Currently unemployed | 0.025 | 0.74 |
| Medical problems prior to March 15^th^ | -0.098 | 0.18 |
| **Exposure Group Membership** |  |  |
| At mosque | 0.160 | 0.03 |
| Wider community | -0.110 | 0.14 |
| Family present, not injured | 0.172 | 0.02 |
| Family present, injured | 0.061 | 0.41 |
| Family shaheed (family member was killed) | -0.021 | 0.78 |
| Survivor, injured | 0.050 | 0.49 |
| Survivor, not injured | -0.006 | 0.93 |
| **Reports of Family Stressors** |  |  |
| Financial concerns | 0.036 | 0.63 |
| Housing problems | -0.051 | 0.49 |
| Immigration issues | -0.136 | 0.06 |
| Employment issues | -0.044 | 0.54 |
| Family tensions | -0.163 | 0.03 |
| Family mental health | -0.026 | 0.72 |
| Legal issues | 0.048 | 0.51 |
| Concerns for children | 0.081 | 0.27 |
| Other concerns | -0.044 | 0.55 |
| **Activities Post-March 15^th^** |  |  |
| Māori community events attendance | 0.149 | 0.04 |
| Muslim social event attendance | 0.138 | 0.06 |
| Islamic Scholar attendance | 0.130 | 0.08 |
| Parent workshop | 0.126 | 0.09 |
| Life coach | 0.148 | 0.04 |
| **Services Post-March 15^th^** |  |  |
| Government disability and financial support | 0.177 | 0.02 |
| Purapura Whetu (Māori support services) | 0.143 | 0.05 |
| Victim support services | 0.133 | 0.07 |
| Police family liaison service | 0.173 | 0.02 |
| Secondary physical health care services | 0.180 | 0.01 |
| Secondary mental health care services | 0.161 | 0.03 |
| Immigration services | 0.141 | 0.06 |
